# Investigating child drowning prevention mechanisms in pioneer countries

**Published:** 2023-12

**Authors:** Bijan Piryaei, Sima Sajadi

**Affiliations:** ^*a* IRSwim, Tehran, Iran. ^

## Abstract

**Background:**

In the last decade, drowning has killed over 2.5 million people throughout the world. The highest rate of drowning is among under-five-year children. Despite efforts made for prevention, child drowning is still a challenge. This study aimed to investigate the drowning prevention strategies implemented in leading countries.

**Methods:**

Available sources were reviewed and the common drowning prevention strategies of different countries were specified. USA, Australia, and Canada were compared based on the following aspects: 1) The parameters presented in the drowning reports, and 2) The considered preventive measures. Finally, the strategies were compared according to these aspects.

**Results:**

Comparing the preventive measures in the strategies adopted by the target countries revealed that the USA considers five monitoring measures including restricting access, water safety training, rescue training, and life jackets, while Australia and Canada do not include life jackets and rescue training, respectively. Examining the parameters presented in the drowning reports of these countries showed that the USA presents the most parameters (i.e. sex, life stages, activity, location, time, fall, alcohol/drugs, life jacket, visitor, swim skills, race, floods/storms, remoteness, diseases, weather, and supervision) in its reports. Among these parameters in Australia, life jacket, visitor, swim skills, race, weather, and supervision, and in Canada, race, floods/storms, remoteness, diseases, and weather were not published.

**Conclusions:**

This study indicates the strategies used in these three countries are generally similar, however, there are some differences in the manner of implementation and details of the considered parameters and preventive measures. Establishing a central organization for drowning prevention in Iran is suggested, which can provide strategies for drowning prevention at a national level according to the guidelines of the World Health Organization and leading countries. Our strategies for drowning prevention should be localized regarding Iran's climate and culture.

**Keywords:**

Drowning, Drowning Prevention, Water Safety

